# Estimating the changing nature of Scotland's health inequalities by using a multivariate spatiotemporal model

**DOI:** 10.1111/rssa.12447

**Published:** 2019-04-09

**Authors:** Eilidh Jack, Duncan Lee, Nema Dean

**Affiliations:** ^1^ University of Glasgow UK

**Keywords:** Bayesian modelling, Disease mapping, Health inequalities, Multivariate spatiotemporal correlation

## Abstract

Health inequalities are the unfair and avoidable differences in people's health between different social groups. These inequalities have a huge influence on people's lives, particularly those who live at the poorer end of the socio‐economic spectrum, as they result in prolonged ill health and shorter lives. Most studies estimate health inequalities for a single disease, but this will give an incomplete picture of the overall inequality in population health. Here we propose a novel multivariate spatiotemporal model for quantifying health inequalities in Scotland across multiple diseases, which will enable us to understand better how these inequalities vary across disease and have changed over time. In developing this model we are interested in estimating health inequalities between Scotland's 14 regional health boards, who are responsible for the protection and improvement of their population's health. The methodology is applied to hospital admissions data for cerebrovascular disease, coronary heart disease and respiratory disease, which are three of the leading causes of death, from 2003 to 2012 across Scotland.

## 1 Introduction

Disease risk is not constant over space and time, and is often impacted by exposure to risk inducing behaviour such as consumption of alcohol. Poverty and, more generally, deprivation are major factors in the spatial variation that is observed in the risk of disease, with more highly deprived areas usually exhibiting elevated levels of disease risk (McCartney, 2012). This difference in disease risk between social groups living in different areas is known as a health inequality (or inequity). Importantly, health inequalities refer to the unfair and avoidable differences in people's health, and are based largely on socio‐economic factors such as income, wealth and education. They are fundamentally driven and shaped by economics, social policy and politics, which in turn lead to an unequal distribution of income, power and wealth. On a global level the world's poorest people tend to have the worst health; for example, the average life expectancy in Japan is 83.7 years, compared with 50.1 years in Sierra Leone (World Health Organization, 2016).

Health inequalities also exist within countries and are seen in countries of all ranges of incomes. There is evidence which shows that individuals who live at the poorer end of the socio‐economic spectrum exhibit poorer health regardless of the wealth of the country (World Health Organization, 2008). The first major report on health inequality in the UK was the Black report (Black *et al*., 1982), which was commissioned by the Labour Government in 1977. The report showed the unequal distribution of ill health and death across the UK and concluded that these inequalities were due mainly to social inequalities affecting health. The Acheson report (Acheson, 1998) confirmed findings from the Black report that the ‘weight of scientific evidence supports a socio‐economic explanation of health inequalities’. Following the publication of these reports it became widely recognized that ‘social class’ had a strong bearing on life expectancy. This is illustrated in Table 2.1 of Bartley (2016) which shows standardized mortality ratios by Registrar‐General's social class in men aged 15–64 years, with a standardized mortality ratio for class I (professional) of 66 compared with 189 for class V (unskilled manual) in 1991. More recently, the Marmot (2010) review's key policy objectives focused on the social determinants of health.

Reports such as these have aided the development of several social models, which differ from statistical models in that the aim is to explain the behaviours of the people who are involved in a certain event or activity. Three of the most common models of explanation for health inequalities, which were described in detail in Bartley (2016), are behavioural, material and psychosocial. Very briefly, behavioural and ‘cultural’ explanations refer to the existence of health inequality due to differences in lifestyle between social groups, most notably smoking, exercising for leisure and quantity of fats, sugars and salt in the diet. The psychosocial model identifies the ‘psychosocial risk factors’ which impact health, including social support, autonomy at work and the balance between home and work (Hemmingway and Marmot, 1999). The materialistic model recognizes the significance of

‘the … diffuse consequences of the class structure: poverty, work conditions … and deprivation in its various forms in the home and immediate environment, at work, in education and the upbringing of children and more generally in family and social life’

(Black *et al*., 1982). Much of the literature attempts to use a combination of these three models when attempting to explain the causes of health inequality.

In this study we focus on Scotland for several reasons. First of all, Scotland has very poor health for a European country, with the lowest and most slowly improving life expectancy compared with all other western European countries (Walsh *et al*., 2016). Scotland also has the widest health inequalities in western Europe (Popham and Boyle, 2011). For example, in 2015 men living in the most affluent areas in Scotland experienced 23.8 more years of ‘good health’ compared with those living in the most deprived areas (22.6 years for women; NHS Health Scotland (2015)). On an individual level, this is a tale of human tragedy, with too many Scots experiencing poor health and dying prematurely as a direct result of health inequalities. These inequalities in health also have huge economic repercussions for Scotland and place an enormous burden on the National Health Service (NHS). For example, it has been estimated that, if the death rate across Scotland fell to the level of the least deprived areas, the economic benefit could exceed £20 billion (Audit Scotland, 2012).

Reducing health inequalities has been a priority for the Scottish Government for many years, and in 2007 they established the Ministerial Task Force for Health Inequalities whose aim was ‘to identify and prioritise practical actions to reduce the most significant and widening health inequalities’ (Scottish Government, 2008). In the current paper we focus in particular on health inequalities between Scotland's 14 regional health boards (HBs), who are responsible for the protection and improvement of their populations’ health. In 2011–2012 the Scottish Government allocated around £170 million to the HBs to address health inequalities directly, giving them direct responsibility for tackling this problem (Audit Scotland, 2012). There have been many reports by official bodies such as the Scottish Government, NHS Scotland and Audit Scotland (Scottish Government, 2008; NHS Health Scotland, 2015; Audit Scotland, 2012) on Scotland's health inequalities, with a focus on ways to reduce these in the future.

On the research side, Taulbut *et al*. (2014) compared west–central Scotland with other post‐industrial regions of Europe, and Leyland *et al*. (2007) examined patterns in, and causes of, inequalities for regions of Scotland. Comparisons between other European countries and Scotland were the focus of Walsh *et al*. (2016), but they concentrated on explaining Scotland's, and particularly Glasgow's, excess mortality. However, this research collectively lacks a detailed analysis of health inequalities for multiple diseases at the small area scale in Scotland, which is the focus of this paper.

There is an extensive literature in modelling spatiotemporal variation in the risk of a single disease from small area data, with many approaches being based on conditional auto‐regressive (CAR) models (Besag *et al*., 1991). Many adaptations of this model have been proposed since, including Bernardinelli *et al*. (1995), who utilized correlated linear time trends for each area. MacNab and Dean (2002) extended this to non‐linear trends, and Knorr‐Held (2000) utilized temporal effects, spatial effects and a space–time interaction. Separately, many models have been proposed for multivariate disease mapping in a purely spatial setting, using multivariate CAR spatial models, including Kim *et al*. (2001), Carlin and Banerjee (2003) and Gelfand and Vounatsou (2003).

However, very few multivariate space–time models have been proposed for modelling the risk of multiple diseases in space and time simultaneously. Examples include Richardson *et al*. (2006), who proposed a shared component model for diseases, which was extended to allow for more than two diseases by Tzala and Best (2008). Alternatives were proposed by Quick *et al*. (2017) using non‐separable multivariate space–time CAR models. Additionally Bradley *et al*. (2015, 2018) proposed a multivariate spatiotemporal mixed effects model which would allow them to model high dimensional data sets efficiently.

This paper adds to this small literature by proposing a novel spatiotemporal multidisease model for quantifying health inequalities in Scotland. Our main focus is answering the following questions of interest.

(a) Are there health inequalities between Scotland's HBs and how are these changing over time and over disease?
(b) Within an HB, how do average levels of risk and temporal trends change between diseases?
(c) How are health inequalities changing over time in small areas in Scotland across multiple diseases?
(d) What effect do the covariates have on risk and how does this change by disease?
(e) Are there some areas which have high risk for all three diseases?

We answer these questions in Section 4, whereas the data and methodology are respectively presented in Sections 2 and 3. Finally, Section 5 provides a discussion of the conclusions drawn and possible future work.

The data that are analysed in the paper and the programs that were used to analyse them can be obtained from

https://rss.onlinelibrary.wiley.com/hub/journal/1467985x/seriesa-datasets

## 2 Scotland study

### 2.1 Study region

The study region is Scotland, UK, which has a population of around 5.3 million. It is split into *n*=1235 non‐overlapping small administrative areas known as intermediate geographies (IGs) (http://www.gov.scot/Publications/2005/02/20732/53083), which contain on average 4000 household residents. The 14 HBs which we are interested in quantifying inequalities between are a range of sizes, both geographically and in terms of population. The HBs with the smallest populations are two of the island boards, Orkney and Western Isles, with 2012 population estimates of 21530 and 23210 respectively. In contrast, Greater Glasgow and Clyde, and Lothian are the most populated HBs despite their relatively small geographical area, with populations of 1217025 and 843733 respectively. This is because both of Scotland's largest cities are in these HBs. Glasgow and Edinburgh respectively. Further details about the HBs are provided in Section 2 of the on‐line supplementary material.

### 2.2 Disease data

The disease data for each IG are yearly counts of the numbers of hospital admissions for three of Scotland's biggest killers (ScotPHO, 2016), namely cerebrovascular disease, coronary heart disease and respiratory disease, for the years 2003–2012 in each IG. In the middle of our time period (2007) the death rates per 100000 for Scotland were 103.2, 180.7 and 142.5 for the three diseases respectively. Coronary heart disease is defined by using the international classification of diseases version 10 codes I20–I25, whereas the codes are J00–J99 and R09.1, and I60–I69 and G45 for respiratory and cerebrovascular disease respectively.

The 10‐year time period was chosen for a few reasons. Firstly, the data for these years are freely available from the Scottish Statistics web site (http://statistics.gov.scot/). Secondly, during this period there were several key policy changes in Scotland which may have either directly or indirectly helped to reduce health inequalities in Scotland. For example, The Smoking Health and Social Care (Scotland) Act 2005 banned smoking in any enclosed public space in Scotland from March 26th, 2006, which is of interest since smoking has been proven to increase the likelihood of an individual developing heart disease, stroke and respiratory disease (US Department of Health and Human Services, 2014).

To adjust for age and sex differences in the populations in each IG, the expected numbers of hospital admissions were calculated separately for each disease by using indirect standardization based on age‐ and sex‐adjusted rates for the whole of Scotland. Given that one of the goals of this analysis is to investigate temporal trends in disease risk, the rates for the year 2006–2007 were used to calculate the expected values for all years. Letting *i* denote IG (*i*=1,…,1235), *t* denote year since 2003 (*t*=1,…,10) and *d* denote disease (*d*=1, cerebrovascular disease; 2, coronary heart disease; 3, respiratory disease), the simplest measure of disease risk is the standardized incidence ratio ${\hat{R}}_{\mathrm{itd}}=Y_{\mathrm{itd}}/E_{\mathrm{itd}}$, where *Y*_*itd*_ is the observed number of hospitalizations and *E*_*itd*_ is the expected number of hospitalizations. Values of the standardized incidence ratio greater than 1 represent elevated levels of disease risk, and values less than 1 correspond to decreased levels of disease risk; for example, a standardized incidence ratio of 1.2 corresponds to a 20% increase in risk.

**Figure 1:**
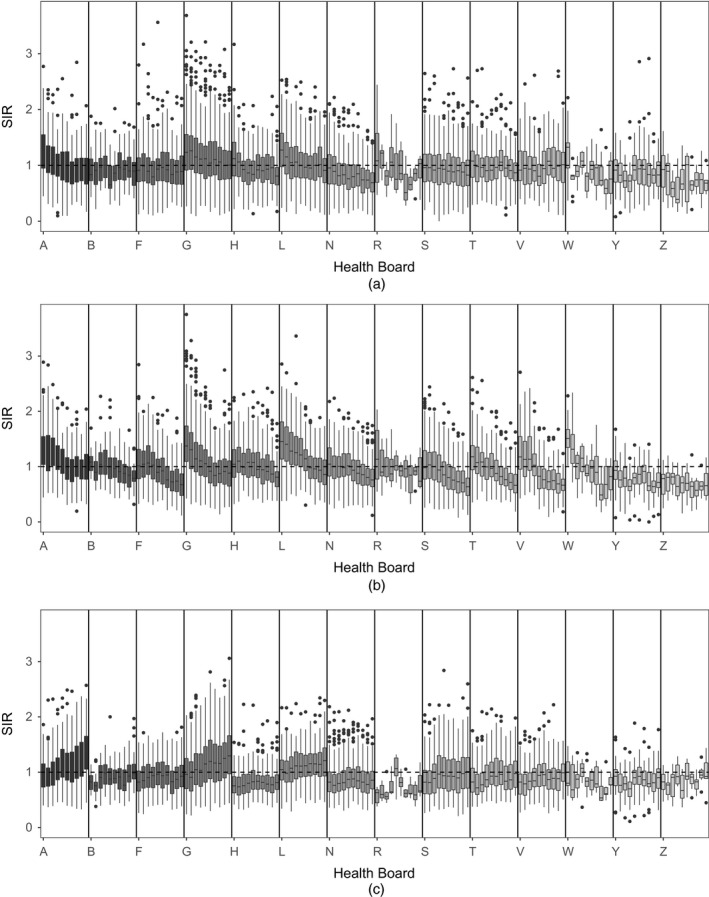
Boxplots of standardized incidence ratios for (a) cerebrovascular disease, (b) coronary heart disease and (c) respiratory disease for IGs in each HB at each year (2003–2012): A, Ayrshire and Arran; B, Borders; F, Fife; G, Greater Glasgow and Clyde; H, Highland; L, Lanarkshire; N, Grampian; R, Orkney; S, Lothian; T, Tayside; V, Forth Valley; W, Western Isles; Y, Dumfries and Galloway; Z, Shetland

To illustrate the temporal trends by HB, which is a key goal of this study, Fig. 1 shows boxplots of the standardized incidence ratio values for the IGs in each HB at each time point for each disease. From this it can clearly be seen that the risk of disease is not constant within an HB over the time period. Similar trends can be seen across some of the HBs in each disease; for example many of the HBs for cerebrovascular disease and coronary heart disease have decreasing trends, whereas for respiratory disease many of the HBs have an increasing trend. However, not all the HBs within a disease follow these general patterns; for example for respiratory disease Highland H, Grampian N, Forth Valley V and Dumfries and Galloway Y all seem to have reasonably constant risks over the time period. This highlights that within an HB the trends are not consistent within a disease, which suggests using HB and disease‐specific trends in the modelling. Finally, the plots show much more variability over time for the three island HBs Orkney R, Western Isles W and Shetland Z, due to the small number of IGs in these HBs.

**Figure 2:**
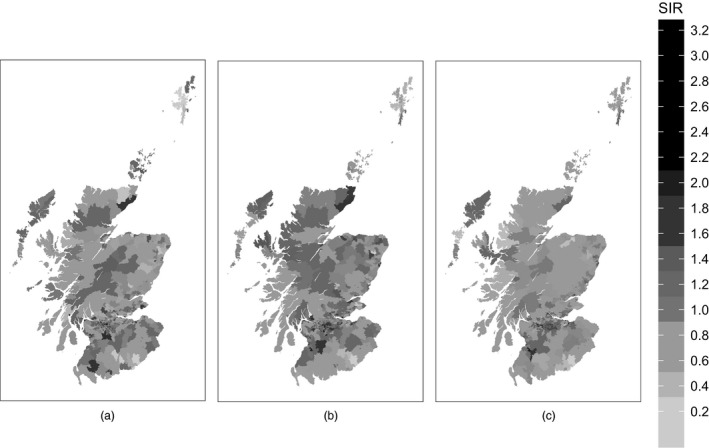
Standardized incidence ratios for (a) cerebrovascular disease, (b) coronary heart disease and (c) respiratory disease for each IG in Scotland in 2006

To assess the presence of spatial variation in the data for each disease, Fig. 2 shows the standardized incidence ratios across IGs in Scotland in 2006 for cerebrovascular disease, coronary heart disease and respiratory disease. From these maps we can see that the spatial patterns for each disease are not the same. Both coronary heart disease and cerebrovascular disease have more areas in northern Scotland with high disease risk than does respiratory disease. This suggests having disease‐specific spatial risk surfaces in the modelling. Animations of these maps for each disease showing the changes over time can be found in section 3 of the on‐line supplementary material.

### 2.3 Covariate data

Potential covariates were identified to help to describe the spatial variation in disease risk across Scotland, which enables us to explore the unexplained excess risk in the HBs after accounting for these known factors. Firstly, the percentage of 16–64‐year‐olds claiming Job Seeker's Allowance is used as a proxy measure of deprivation, since it is well known that higher levels of socio‐economic deprivation are linked to increased risk of disease (Audit Scotland, 2012). Given that there is also evidence that a person's ethnicity can have an influence on the risk of certain diseases (Scottish Government, 2012), the percentage of the population of Asian ethnicity and the percentage of the population of black ethnicity are also included as potential explanatory variables. Both of these covariates are highly skewed to the right with many nearly zero values, and so a log‐transformation was applied. Finally, an urban–rural factor was included using the Scottish Government's urban–rural twofold classification which can be found at http://www.gov.scot/Topics/Statistics/About/Methodology/UrbanRuralClassification. This was chosen as an indication of access to hospitals, as perhaps those who live in rural areas are less likely to be admitted to hospital if they live in remote areas where hospitals are difficult to reach.

### 2.4 Exploratory analysis

To assess the presence of residual spatial correlation in the data, a Poisson generalized linear model was fitted to the data for 2003 for each disease separately, with the covariates described. Moran's *I* (Moran, 1950) statistics were then calculated by using the residuals from these models, and the results show that for all three diseases strong spatial correlation was present, with Moran's *I*‐statistics of 0.107, 0.227 and 0.241 for cerebrovascular, coronary heart and respiratory disease respectively, with significant associated *p*‐values less than 0.001 for all three statistics.

Pairwise correlations were also calculated between the residuals for each disease, with a correlation of 0.211 between cerebrovascular and respiratory disease, 0.216 between cerebrovascular and coronary heart disease and 0.337 between coronary heart and respiratory disease. This indicates that there is some residual between‐disease correlation in the data, suggesting that between‐disease correlation needs to be modelled. To investigate whether disease‐specific covariate effects would be appropriate, the estimated covariate effects from the same Poisson generalized linear models as before were checked and some differences in these were found. For example the covariate log(% of population of Asian ethnicity) showed a significant protective effect for coronary heart disease, a significant increased risk effect for respiratory disease and no significant effect for cerebrovascular disease. It is therefore appropriate to include separate covariate effects for each disease.

Finally, to assess the presence of temporal correlation, the average lag 1 correlation coefficient was calculated for each disease across the IGs, but given that we have a very short time series (only 10 time points) the results from this were inconsistent. However, given that the data come from the same group of people every year, *a priori*, we would expect there to be temporal correlation and so we shall account for this in the final model.

## 3 Methodology

We propose a novel multivariate spatiotemporal Bayesian hierarchical model for these data, with the aim of quantifying how health inequalities have changed over time in Scotland at the HB level.

### 3.1 Likelihood model

The first level of the hierarchical model that we specify is given by

(1) $$\begin{array}{cc} & Y_{\mathrm{itd}}∼\text{Poisson}\left(E_{\mathrm{itd}}R_{\mathrm{itd}}\right),i=1,\ldots ,n\;\left(=1235\right),t=1,\ldots ,T\;\left(=10\right),d=1,2,D\;\left(=3\right),\\ & \ln\left(R_{\mathrm{itd}}\right)=x_{i}^{T}\beta_{d}+H_{h(i)td}+\phi_{\mathrm{id}},h\left(i\right)=1,\ldots ,H\;(=14), \end{array}$$

where *Y*_*itd*_ and *E*_*itd*_ are the observed and expected numbers of hospital admissions in IG *i*, at time point *t* for disease *d*, whereas *R*_*itd*_ is the risk relative to the expected numbers *E*_*itd*_. We model the log‐risk with three components, the first of which is the *p*×1 vector of known covariates **x**_*i*_=(1,*x*_*i*2_,…,*x*_*ip*_), including an intercept term, with disease‐specific regression parameters ***β***_*d*_ = (*β*_1*d*_,…,*β*_*pd*_). Given that we do not have access to temporally varying covariate information we cannot include this in the model. However, we did consider allowing the regression parameters to vary over time and disease, i.e. ***β***_*td*_ = (*β*_1*td*_,…,*β*_*ptd*_), but the parameter estimates showed little change over time and can be found in Section 6.1 of the on‐line supplementary material. The prior that was specified is ***β***_*d*_∼*N*(0,100*I*), where *I* is a *d*×*d* identity matrix, which is weakly informative to allow their values to be informed by the data. The remaining two components are a baseline disease‐specific spatial effect *ϕ*_*id*_, and a disease‐ and HB‐specific temporal trend $H_{h(i)td}$, where *h*(*i*) denotes that IG *i* is within HB *h*. We have chosen not to include temporal variation in the random effects *ϕ*_*id*_, because we want all temporal variation to be incorporated in the temporally varying HB effects as these are of key interest.

### 3.2 Disease‐specific spatial effects

In Section 2.4, we found evidence of substantial residual spatial correlations in the data, which we model via disease‐specific spatial random effects. Spatial correlation is induced into these random effects via the spatial neighbourhood matrix *W*, which is an *n*×*n* binary matrix, where *w*_*ij*_=1 if two areas share a common border and *w*_*ij*_=0 if not. Also *w*_*ii*_=0 for all *i*. However, there are several island IGs that have no defined neighbours by using this specification (six in total), and these were assigned a single neighbour based on the closest IG.

Since there was evidence from Fig. 2 that a common spatial surface may not be appropriate for all diseases, we model the multidisease spatial effects *ϕ* by a multivariate version of the Leroux CAR prior (Leroux *et al*., 2000) given by

(2) $$\begin{array}{cc} & \phi_{i}|\phi_{-i}∼N(\frac{\rho \sum_{j=1}^{n}w_{\mathrm{ij}}\phi_{j}}{\rho \sum_{j=1}^{n}w_{\mathrm{ij}}+1-\rho },\frac{1}{\rho \sum_{j=1}^{n}w_{\mathrm{ij}}+1-\rho }\Sigma ),\Sigma ∼\text{Inverse‐Wishart}\left(3,I\right),\\ & \rho ∼\text{Unif}(0,1), \end{array}$$

where ***ϕ***_*i*_=(*ϕ*_*i*,1_,…,*ϕ*_*i*,*D*_) and *ϕ*_−*i*_=(***ϕ***_1_,…,***ϕ***_*i*−1_,***ϕ***_*i*+1_,…,***ϕ***_*n*_). The parameter *ρ* controls the level of spatial correlation in the data, with *ρ*=0 corresponding to independence in space and *ρ*=1 corresponding to the multivariate extension of the intrinsic CAR prior (Besag *et al*., 1991). Disease‐specific spatial correlations ***ρ***=(*ρ*_1_,*ρ*_2_,*ρ*_3_) were considered, but analyses on each disease separately suggested that a single *ρ*‐parameter was sufficient. The covariance matrix Σ is included to allow for between‐disease correlation. Given that there is no particular reason for an *a priori* structure for this matrix, a conjugate inverse Wishart prior is assigned to Σ, with weakly informative hyperparameters to allow these parameters to be estimated mainly by the data.

### 3.3 Temporally varying health board effects

A key question in our analysis is to investigate the health inequalities between Scotland's 14 regional HBs, and how these change over time and between disease. Therefore we include disease‐specific HB temporal trends in the model, $H_{\mathrm{hd}}=\left(H_{h1d},\ldots ,H_{\mathrm{hTd}}\right)$, which are modelled by the first‐order auto‐regressive process

(3) $$H_{\mathrm{htd}}∼N\left(\alpha_{d}H_{h,\;t-1,\;d},\sigma_{d}^{2}\right),\sigma_{d}^{2}∼\text{Inverse‐Gamma}\left(0.001,0.001\right),\alpha_{d}∼\text{Unif}\left(0,1\right),$$

where $H_{\mathrm{htd}}$ is the effect for HB *h* at time point *t* for disease *d*. Temporal correlation is induced via the hyperparameter *α*_*d*_, with *α*_*d*_=0 indicating independence across time whereas *α*_*d*_=1 indicates strong temporal dependence. Initially this model allowed the hyperparameters *α*_*d*_ and $\sigma_{d}^{2}$ to vary by disease and HB; however, in this case the parameters were not well identified by the data, and so *α*_*d*_ and $\sigma_{d}^{2}$ varied by disease only. As before, weakly informative priors were assigned to *α*_*d*_ and $\sigma_{d}^{2}$ to allow their values to be mainly informed from the data.

### 3.4 Inference and software

To obtain posterior summaries of each parameter, samples were drawn from the posterior distribution by using Markov chain Monte Carlo simulation using both Gibbs sampling and Metropolis steps. The Markov chain Monte Carlo algorithm was written in R (R Development Core Team, 2014). However, because of the large number of random effects that must be sampled at each iteration, this part of the estimation, along with the update for the HB effects, was implemented by using C×× via the R package Rcpp (Eddelbuettel and François, 2011; Eddelbuettel, 2013). Additionally, as *W* is sparse, we utilized its triplet form to speed up computation. To make this research reproducible the code and data are also available from https://github.com/eilidhjack/MVSTsoftware.

## 4 Results

The multivariate spatiotemporal model that was proposed in Section 3 was applied to the data that were described in Section 2. Inference is based on a single Markov chain Monte Carlo chain with 150000 iterations, 50000 of which were discarded for the burn‐in period. The chain was thinned by 5 because of limitations in computer memory and to make the samples closer to independent, and so the posterior estimates are based on 20000 samples. Convergence was checked both by examining parameter trace plots and by Geweke diagnostics (Geweke, 1992).

The posterior medians and 95% credible intervals for the spatial and temporal correlation parameters are shown in Table 1. The posterior median estimate for the spatial correlation parameter *ρ* is 0.432, which suggests a moderate level of spatial correlation across Scotland for the three diseases. A separate temporal correlation parameter *α*_*d*_ was estimated for each disease, and Table 1 shows similar estimates for coronary heart disease and respiratory disease, with posterior medians of 0.870 and 0.833 respectively. Although the posterior median for cerebrovascular disease is not as high (0.689), it still shows that the data contain moderate levels of temporal correlation.

**Table 1:** Estimates and 95% credible intervals for spatial, temporal and between‐disease correlations

*Correlation*	*Posterior*	*95% credible*
	*median*	*interval*
*Spatial*		
*ρ*	0.432	(0.360, 0.512)
*Temporal*		
*α*—cerebrovascular disease	0.689	(0.562, 0.799)
*α*—coronary heart disease	0.870	(0.790, 0.946)
*α*—respiratory disease	0.833	(0.720, 0.940)
*Between‐disease*		
Cerebrovascular and coronary heart disease	0.498	(0.465, 0.525)
Cerebrovascular and respiratory disease	0.559	(0.533, 0.578)
Coronary heart and respiratory disease	0.645	(0.633, 0.658)

The posterior correlation between diseases is calculated via the covariance matrix Σ and, for example, the correlation between coronary heart disease and respiratory disease is calculated as

(4) $$\Sigma_{12}/\surd \left(\Sigma_{11}\Sigma_{22}\right).$$

Table 1 shows the posterior medians of the between‐disease correlations, along with the 95% credible intervals. All pairs of diseases show moderate correlation, with the correlation between coronary heart disease and respiratory disease being the strongest (0.645), whereas the correlation between coronary heart disease and cerebrovascular disease is the weakest (0.498).

### 4.1 Health board effects

**Figure 3:**
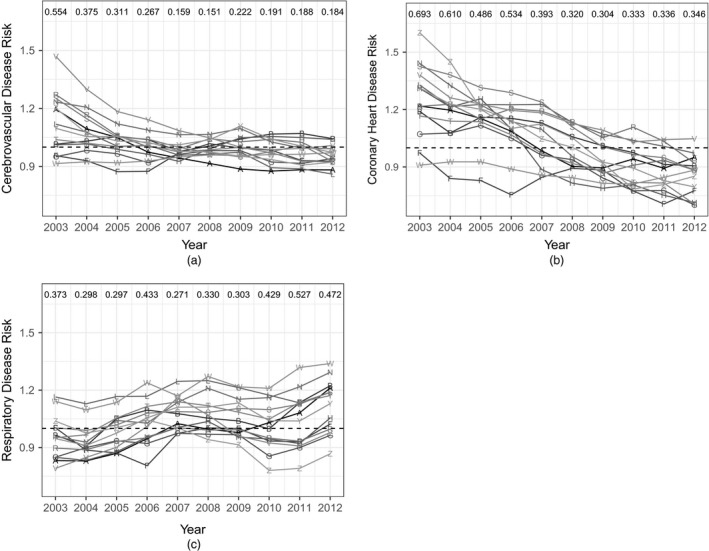
HB risk effects (posterior medians) across time ($R_{\mathrm{htd}}=\Sigma_{i\in I_{h}}\pi_{i}\exp\left(\phi_{\mathrm{id}}+H_{\mathrm{htd}}\right)/\Sigma_{i\in I_{h}}\pi_{i}$) (the numbers at the top of each graph represent the range in the median HB effects for each year) (A, Ayrshire and Arran; B, Borders; F, Dumfries and Galloway; G, Fife; H, Forth Valley; L, Grampian; N, Greater Glasgow and Clyde; R, Highland; S, Lanarkshire; T, Lothian; V, Orkney; W, Shetland; Y, Tayside; Z, Western Isles): (a) cerebrovascular disease; (b) coronany heart disease; (c) respiratory disease

To investigate whether there are health inequalities between Scotland's 14 regional HBs within each disease and how these are changing over time (question (a), Section 1), Fig. 3 shows the risk that is associated with each HB for each disease separately, after adjusting for the known covariates. The risk that is associated with the HBs is defined as

$$R_{\mathrm{htd}}=\frac{\sum_{i\in I_{h}}\pi_{i}\exp\left(\phi_{\mathrm{id}}+H_{\mathrm{htd}}\right)}{\sum_{i\in I_{h}}\pi_{i}},$$

where *I*_*h*_ is the set of all IGs *i* belonging to an HB *h* and *π*_*i*_ is the proportion of Scotland's population living in IG *i*. For all diseases it can be seen that there are health inequalities between the HBs, as there are differences between the estimated HB posterior medians within each disease. The risk of disease is not consistent between HBs; nor is it constant over time. For cerebrovascular disease we see a general decreasing trend, and after around 2007 the trends seem to level off. We also see a narrowing of the inequality between the HBs with a difference of 0.554 between the highest and lowest median HB risk in 2003 compared with 0.184 in 2012. Given that the island boards have significantly fewer IGs than the mainland boards, we tend to see greater variation in risk estimates for these boards over the time period. However, even when ignoring these boards, there is still a reduction in the inequality between HBs from 0.301 in 2003 to 0.184 in 2012.

For coronary heart disease a much stronger decreasing trend can be seen over almost all the HBs compared with cerebrovascular disease. We also see a narrowing of the inequality between the HBs, with a range of medians in 2003 of 0.693 compared with 0.346 in 2012. After removing the island boards the range in inequality lessens from 0.468 in 2003 to 0.267 in 2012.

Finally, the HB effects for respiratory disease do not show the same pattern as the previous two diseases. In general, most HB risks seem to go up over the time period. We also see a widening in health inequalities between the HBs for this disease, with the range between medians increasing from 0.373 in 2003 to 0.472 in 2012. However, once the island boards have been removed, the change in inequality is almost non‐existent from 0.333 to 0.329.

**Figure 4:**
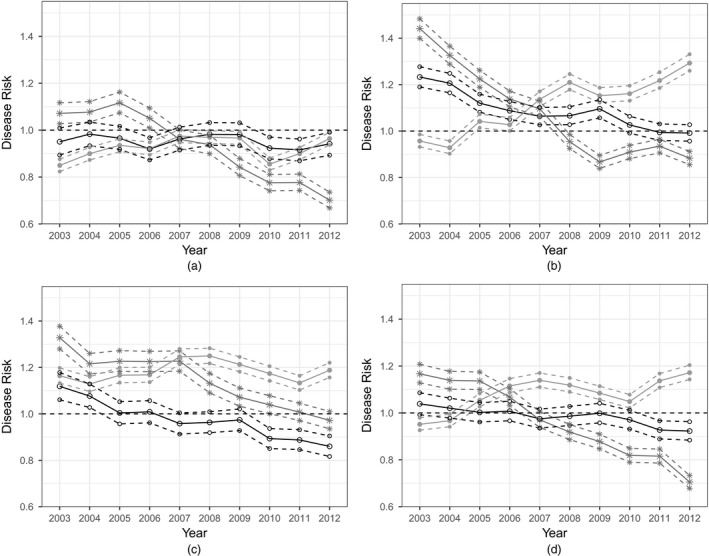
HB risk effects (posterior medians and 95% credible intervals) across time ($R_{\mathrm{htd}}=\Sigma_{i\in I_{h}}\pi_{i}\exp\left(\phi_{\mathrm{id}}+H_{\mathrm{htd}}\right)/\Sigma_{i\in I_{h}}\pi_{i}$) for each disease for HBs (a) Fife, (b) Greater Glasgow and Clyde, (c) Grampian and (d) Lothian: 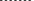, null risk of 1; ∘, cerebrovascular disease; *, coronary heart disease; 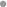, respiratory disease

We are also interested in comparing how average HB levels and temporal trends change between diseases within an HB (question (b), Section 1). To answer this, Fig. 4 shows the posterior medians and 95% credible intervals for the HB effects across time for each disease for the HBs Fife, Greater Glasgow and Clyde, Grampian and Lothian. (Plots for all 14 HBs can be found in section 4 of the on‐line supplementary material.) There are some HBs where the difference in risk between the diseases is not large. For example, Fife shows a similar risk of cerebrovascular and respiratory disease, with posterior medians below 1 and remaining reasonably constant over the years. However, coronary heart disease risk shows a decreasing trend with an average risk and 95% credible intervals entirely above 1 at the start of the time period (and higher than the other two diseases) but below 1 at the end (and now lower than the other two diseases).

For the other HBs the differences in risk between the diseases is much more obvious. For example, for Greater Glasgow and Clyde there is a very strong decrease in risk of coronary heart disease until around 2009 when it levels off, a less steep decreasing trend for cerebrovascular disease, but an increasing trend can be seen for respiratory disease, with risk increasing from the lowest of all diseases in 2003 to the highest in 2012. Similar trends can be seen for Lothian, although the magnitudes of the trends are not consistent with Greater Glasgow and Clyde. Another interesting feature is that the same general trend for each disease is not seen over all the HBs. For example, although Grampian does show a decreasing trend for cerebrovascular and coronary heart disease, the difference in the strength of this decrease is not greatly different from each other although coronary heart disease shows a much stronger decreasing trend compared with cerebrovascular disease for the other three HBs that are shown here. In fact, Grampian is the only HB whose risk of coronary heart disease is still higher in 2012 than the risk of cerebrovascular disease. The trend for respiratory disease in Grampian also does not follow the general increasing trend, with only a very small increase in risk from 2003 to 2012.

### 4.2 Overall health inequalities

**Figure 5:**
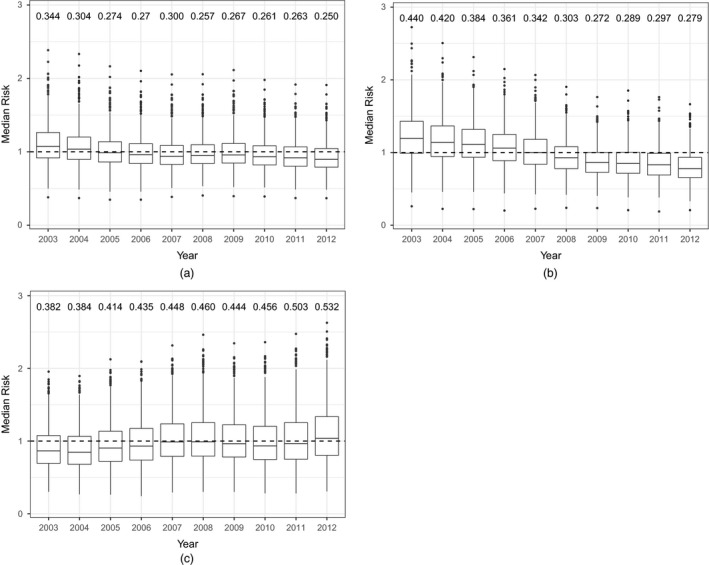
Boxplots of disease risk for (a) cerebrovascular disease, (b) coronary heart disease and (c) respiratory disease in IGs in Scotland from 2003 to 2012: the IQRs across IGs are printed above each boxplot; outliers are those observations that lie outside 1.5 IQR

To investigate whether overall health inequalities have changed over time across the IGs in Scotland (not just between HBs as in Section 4.1) (question (c), Section 1), Fig. 5 shows boxplots of the posterior median disease risk for all IGs for each disease. Printed above each boxplot is the interquartile range IQR across all IGs. For cerebrovascular disease we see a general decrease in the overall trend and in health inequality, which can be seen from the decreasing IQRs, from 0.344 in 2003 to 0.250 in 2012. Similarly, the overall risk of coronary heart disease is decreasing over time, more quickly at the beginning of the time period and after around 2009 this decrease shows signs of levelling off. When looking at the IQRs we can see a decrease in health inequality in coronary heart disease risk, from 0.440 in 2003 to 0.279 in 2012, which again is more noticeable in the period from 2003 to 2009, after which it levels off. Conversely, for respiratory disease, we see the opposite effect. Not only is the overall risk increasing over time, but also the inequality is growing worse, which can be seen from the increase in IQR from 0.382 in 2003 to 0.532 in 2012.

### 4.3 Covariate effects

To assess the effect that covariates have on risk and how this changes over disease (question (d), Section 1), Table 2 shows the point estimates (posterior medians) and 95% credible intervals on the relative risk scale. For cerebrovascular disease the median relative risk RR for the percentage of 16–64‐year‐olds claiming Job Seeker's Allowance is around 1.060 for a 1% increase; so the risk of cerebrovascular disease in an IG increases by 6.0% as the percentage claiming Job Seeker's Allowance increases by 1%. The effect of this covariate for coronary heart disease is similar (RR = 1.065), whereas the effect for respiratory disease is much larger with an increase of around 10.5%. This could be because smoking is one of the main causes of respiratory disease, with nearly eight out of 10 chronic obstructive pulmonary disease deaths deemed to be a result of smoking (Centers for Disease Control and Prevention, 2014), and deprived areas exhibit higher levels of smoking compared with affluent areas (NHS Scotland, 2003a).

**Table 2:** Relative risk estimates RR for a 1% or 1‐unit increase in each covariate (not urban or rural covariate) and 95% credible intervals for the covariates in the model†

*Covariate*	*Median RR*	*95% credible interval*
*Cerebrovascular disease*		
% 16–64‐year‐olds claiming Job Seeker's Allowance	*1.060*	(*1.056, 1.065*)
Log(% Asian)	0.998	(0.985, 1.011)
Log(% black)	*1.008*	(*1.002, 1.015*)
Rural area	0.977	(0.949, 1.006)
*Coronary heart disease*		
% 16–64‐year‐olds claiming Job Seeker's Allowance	*1.065*	*(1.059, 1.070)*
Log(% Asian)	*0.965*	*(0.951, 0.980)*
Log(% black)	1.001	(0.995, 1.008)
Rural area	*0.953*	(*0.924, 0.983*)
*Respiratory disease*		
% 16–64‐year‐olds claiming Job Seeker's Allowance	*1.105*	*(1.098, 1.112)*
Log(% Asian)	0.985	(0.968, 1.001)
Log(% black)	0.992	(0.985, 1.000)
Rural area	0.997	(0.966, 1.033)

†Covariates where the 95% credible intervals do not contain 0 are highlighted in italics.

The covariate log(% of population of Asian ethnicity) showed no evidence of a relationship with cerebrovascular or respiratory disease risk. However, for coronary heart disease the median RR‐estimate is 0.965, suggesting that there may be a very small decrease in coronary heart disease risk as this covariate increases. This is in line with findings from a Scottish Government report (Scottish Government, 2012), which found that those of Chinese ethnicity were the least likely to be diagnosed with cardiovascular disease (which includes coronary heart disease) compared with the national average. In that report those in Indian and Pakistani ethnic groups showed no difference compared with the national average. In this study, the percentage of population of Asian ethnicity includes all Asian ethnic groups, which could explain the small protective effect for this covariate.

Neither coronary heart disease nor respiratory disease shows any evidence of a relationship between disease risk and log(% of population of black ethnicity). However, log(% of population of black ethnicity) was found to have a small detrimental effect of cerebrovascular disease risk, with risk increasing by 0.8% as log(% of population of black ethnicity) increased by 1%. This is line with findings that people of black origin are at higher risk of stroke compared with white people (Stroke Association, 2016).

Finally, there is no evidence that living in a rural or urban area made any difference to the risk that is cerebrovascular or respiratory disease, but for coronary heart disease the risk that is associated with urban areas compared with rural areas is 1/0.953=1.049, i.e. there is an estimated increased risk of coronary heart disease of 4.9% when living in an urban area compared with a rural area.

To assess the sensitivity of the results from this model to the choice of covariates, the model was also run with no covariates and the results in terms of risk estimates were practically identical. Some comparative figures can be found in section 6.2 of the on‐line supplementary material.

### 4.4 Top intermediate geographies risks

It was of interest to identify which IGs showed the highest risk for each disease, and hence if there were any which exhibited high risk for more than one disease. We identified the top five highest risk IGs in 2003 and then in 2012 for all three diseases, and when we compared these IGs across the diseases we note some similarities. For example, the IG Paisley Ferguslie which is in Renfrewshire (south‐west of Glasgow) not only appears in the top five highest risks for all diseases in both years but also actually comes out on top for coronary heart disease and respiratory disease and is second highest for cerebrovascular disease in both years. The IG North Barlanark and Easterhouse South in Glasgow appears in the top five for cerebrovascular disease and coronary heart disease for both years. Finally, Drumchapel North in Glasgow appears for respiratory disease in 2012 as well as for cerebrovascular disease in both years. These areas which have extremely high risk for two or all three diseases show the extent of the health inequality that is experienced in these areas. Ultimately this means that, for the people who live in these places, the risk of hospitalization from any one of these three diseases is much higher than average. A table listing these IGs along with posterior medians and 95% credible intervals for their risks is available in section 5 of the on‐line supplementary material.

### 4.5 Model comparison

To compare our model with an existing model in the literature we decided to fit the model that was proposed by Quick *et al*. (2017) to our data. Similarly to our model this model was designed for multivariate spatiotemporal data; however, unlike our model where the spatial component and temporal component are built separately, this model allows for spatiotemporal dependence in the data by using a single set of random effects. The model is as follows:

(5) $$\begin{array}{cc} & Y_{\mathrm{itd}}∼\text{Poisson}\left(E_{\mathrm{itd}}R_{\mathrm{itd}}\right),\\ & \ln\left(R_{\mathrm{itd}}\right)=x_{i}^{T}\beta_{d}+Z_{\mathrm{itd}}+\phi_{\mathrm{itd}}, \end{array}$$

where $Z_{\mathrm{itd}}$ is a spatiotemporal random effect which also accounts for between‐disease correlation, and $\phi_{\mathrm{itd}}∼N\left(0,\tau_{d}^{2}\right)$. The results from this model were broadly similar to ours, which can be seen in section 6.3 of the on‐line supplementary material. Because of the added complexity of the model that was proposed by Quick *et al*. (2017) and therefore the large number of extra parameters (an effective number of parameters *P*_D_ of 11294 compared with 3045 for our model) the computational time was far greater than for ours. Given that our aim was quantifying HB inequalities, our model was more appropriate in this context.

## 5 Discussion

In this paper a multivariate spatiotemporal model was proposed to estimate health inequalities in Scotland and how they have changed over time. The model included separate covariate effects for each disease, disease‐specific spatial effects and disease‐ and HB‐specific temporal trends. The model was applied to yearly hospital admissions data at the IG level for three of Scotland's biggest killers: cerebrovascular disease, coronary heart disease and respiratory disease for the period 2003–2012.

The main results of this study are that there has been a decrease in risk for cerebrovascular and coronary heart disease across the HBs, but this is accompanied by an increase for respiratory disease. We also found that, even after the covariate effects have been removed, there are still inequalities in disease risk between the HBs for all three diseases. These inequalities change over time, and overall they appear to be narrowing for cerebrovascular disease and coronary heart disease, with a reduction in the range of the median HB risks, ignoring the island HBs, between the first and last time points of 0.142 and 0.191 for each disease respectively. However, these inequalities show no change for respiratory disease with the corresponding difference being only 0.001.

Overall across the IGs in Scotland we found that health inequalities still exist to quite a considerable extent and, although there has been a narrowing for cerebrovascular and coronary heart disease, the inequalities in respiratory disease appear to be growing worse over the time period that is studied here. Research into the prevalence of respiratory diseases in the UK by the British Lung Foundation has shown that in 2011 approximately 67% of UK hospital admissions from respiratory diseases were due to pneumonia, chronic obstructive pulmonary disease and acute lower respiratory infections. From these, both pneumonia and more so chronic obstructive pulmonary disease have shown increasing numbers of diagnoses over the time period 2004–2012 (British Lung Foundation, 2013). This increased risk may be partially due to increased diagnoses, which in turn will bring about an increase in hospital admissions. The increase in respiratory disease admissions could also be due to a reduction in competing causes for hospitalization, given that there has been a reduction in risk of hospitalization for cerebrovascular disease and coronary heart disease. However, this increase in hospital admissions does not occur uniformly across all IGs. Instead, we are seeing a greater increase in numbers in IGs which already had high risks of respiratory disease, which is driving the increase in the health inequality for this disease. For example, in 2003 the estimated risk for Langholm and Canonbie (lowest risk IG) was 0.302, which increased to 0.307 in 2012. In comparison, the estimated risk in 2003 for Paisley Ferguslie (highest risk IG) was 1.955, which increased to 2.627 in 2012.

There were many significant changes to the structure of the health service in Scotland in the years before and during the time period of our data, which could help to explain some of the improvements that were observed. In 1997, ‘Designed to care: renewing the NHS in Scotland’ (Scottish Office, 1997) was published with the main aim of phasing out the internal market, integrating services to eliminate duplication and wasteful competition and merging 47 Scottish trusts into 28. Following the devolution in 1999, ‘Our national health: a plan for action, a plan for change’ (Scottish Executive, 2000) stated that the health budget in Scotland was due to rise from £4.9 billion in 1999–2000 to £6.7 billion in 2003–2004 (which coincides with the start of our time period). This considerable increase in resources was to be used to build a modernized health system and to improve the health of Scotland's population.

In the period 2003–2006 there were several more policy changes which may have contributed to the results that are found in this paper. First of all in 2003, ‘Partnership for care: Scotland's health White Paper’ (NHS Scotland, 2003b) was released, which led to the abolition of the NHS Trusts in 2004 which were absorbed into the HBs which we have focused on in this paper. The HBs were given the single tier of governance and accountability and health improvement was made a priority. This policy change also led to the creation of 40 community health partnerships which were the vehicle for the planning and delivery of primary and community‐based services (Scottish Government, 2010). The community health partnerships were given the responsibility (along with the HBs) to improve health and to reduce health inequalities and could work locally, not only to tackle smoking, obesity, drug and alcohol misuse etc., but also to work with other agencies to tackle many of the other social determinants of health.

The national service framework (Scottish Executive, 2005) set out long‐term plans for the NHS in Scotland over the next 20 years. The key message was to look to the population of Scotland to take more responsibility for their own health and to ‘anticipate and prevent rather than react’. Another key piece of legislation during this time period was The Smoking Health and Social Care (Scotland) Act 2005, which banned smoking in any enclosed public space in Scotland from March 26th, 2006. The ban was described by the Chief Medical Officer Mac Armstrong as bringing ‘far and away the most important improvement in our health in a generation’. In 2007, towards the end of our time period, ‘Better health, better care: action plan’ (Scottish Government, 2007) was introduced, which viewed patients and the public as ‘partners rather than recipients of care’. This again outlines the focus of helping the public to improve their health, acting proactively rather than reactively, particularly in disadvantaged communities. This shift in perspective is crucial to tackling the health inequalities that still exist in Scotland truly.

Reducing the size of Scotland's health inequalities has clearly been a key focus of both the Scottish Government and NHS Scotland, with many of these policy changes directly stating that an improvement in health inequalities as well as overall health is of importance for Scotland's people in future. This paper quantifies these changes, and a concerning feature of our results is the large number of outliers with high risk estimates in Fig. 5. This further highlights the huge problem that Scotland faces in their inequality in overall health and that more needs to be done to target areas which are experiencing much higher risks of disease than the rest of Scotland.

A common problem in areal unit data of this type is that often there are changes to boundaries during the time period for which data are available. For example, in 2014 the Scottish Government released a redrawn version of the IG boundaries and several data sets are publicly available for which the time period overlaps this boundary change. Using data from before and after this change would lead to incomparable inference due to spatial misalignment in the data. This issue could be overcome by utilizing a common latent spatial grid scale and using a data augmentation approach to estimate the data on this scale. Another area for future work would be to consider a clustering‐based modelling approach, to identify areas exhibiting elevated disease risks across multiple diseases.

## Supporting information

‘Web‐based supporting materials for Estimating the changing nature of Scotland's health inequalities using a multivariate spatio‐temporal model’.
