# Supplementary material for: Estimating the changing nature of Scotland's health inequalities by using a multivariate spatiotemporal model
Source: J R Stat Soc Ser A Stat Soc. 2019 Apr 9;182(3):1061–80. doi: 10.1111/rssa.12447 (PMC6563432; doi:10.1111/rssa.12447)
Supplement: Supplementary file 1 — ‘Web‐based supporting materials for Estimating the changing nature of Scotland's health inequalities using a multivariate spatio‐temporal model’. [file RSSA-182-1061-s001.pdf]

Web-based supporting materials for *Estimating the changing nature of Scotland's health inequalities using a multivariate spatio-temporal model*

Eilidh Jack, Duncan Lee, Nema Dean

1. Introduction

The supplementary material contains the following sections. Section 2 provides some additional information on Scotland's 14 health boards (HB). Section 3 provides animations of the standardised incidence ratios (SIR's) for each disease across Scotland over the 10-year time period. Section 4 shows the estimated HB risks across time for each disease for all 14 HB's. Section 5 highlights on the intermediate geographies (IG's) with the highest estimated risks across the 3 diseases. Section 6 provides results from three alternative models and compares these to the model presented in the main paper.

2. Health Boards

A map of Scotland displaying the major urban areas and the geographical extent of the 14 HB's is displayed in Figure 1. The estimated population and number of IG's in each HB is detailed in Table 1. These show that a large land area does not imply a large population, for example the Highland HB is clearly the largest geographically however, is only the 8th largest in terms of population. Whereas the HB Greater Glasgow and Clyde is small in area but has the largest population. Instead, the number of IG's is driven by the population size rather than geographical size, with Greater Glasgow and Clyde having the most IG's of all the HB's. Finally, we can see from the map that Scotland's two biggest cities, Glasgow and Edinburgh are situated in the HB's Greater Glasgow and Clyde and Lothian respectively.

Health Board	Code	Estimated population (2012)	# of IG's
Ayrshire and Arran	A	373,189	92
Borders	B	113,707	29
Fife	F	366,219	103
Greater Glasgow and Clyde	G	1,217,025	272
Highland	H	319,811	76
Lanarkshire	L	572,520	137
Grampian	N	573,420	128
Orkney	R	21,530	6
Lothian	S	842,733	177
Tayside	T	411,749	90
Forth Valley	V	299,099	74
Western Isles	W	27,560	9
Dumfries and Galloway	Y	150,828	35
Shetland	Z	23,210	7

Table 1: Information on Scotland's 14 Health Boards.

3. SIR maps

In order to show the changing spatial patterns in SIR over time, animations of the SIR maps for each disease across time are available to download from `https://github.com/eilidhjack/MVST-software`. For cerebrovascular disease, we see that over time the SIR generally decreases across Scotland, with far more areas with SIR's of less than 1 towards the end of the time period. A similar pattern can be seen for coronary heart disease, with many areas having lower SIR's at the end of the time period than the beginning. Although the SIR values for respiratory disease increase over the time period, the change in pattern is more difficult to see as most of this change happens in the densely populated 'central belt', which can be identified on the map as the area with a large number of small IG's in the lower-middle of the country.

4. Health board effects

Figures 2 and 3 show the temporal trends in the HB effects, R_{htd}, for each disease for all 14 HB's. The posterior medians (solid) and 95% credible intervals (dashed) for cerebrovascular disease are shown in red, green for coronary heart disease and blue for respiratory disease. There are some HB's whose risk for each of the three diseases is reasonably similar over the 10 years. For example, the disease risks for Borders move around the null risk of 1 and don't show huge change over time. For Fife, although at the start of the time period, the posterior median for coronary heart disease was slightly above the null risk of 1, by the end, all three diseases have risk estimates of less than 1. Finally, for Dumfries and Galloway, although there is more variability at the end of the time period, the risks for all three diseases are almost always below 1 and are not hugely different across diseases. However from the plots it can be seen that there are differences in disease risk and patterns over time between the diseases for the rest of the HB's. For

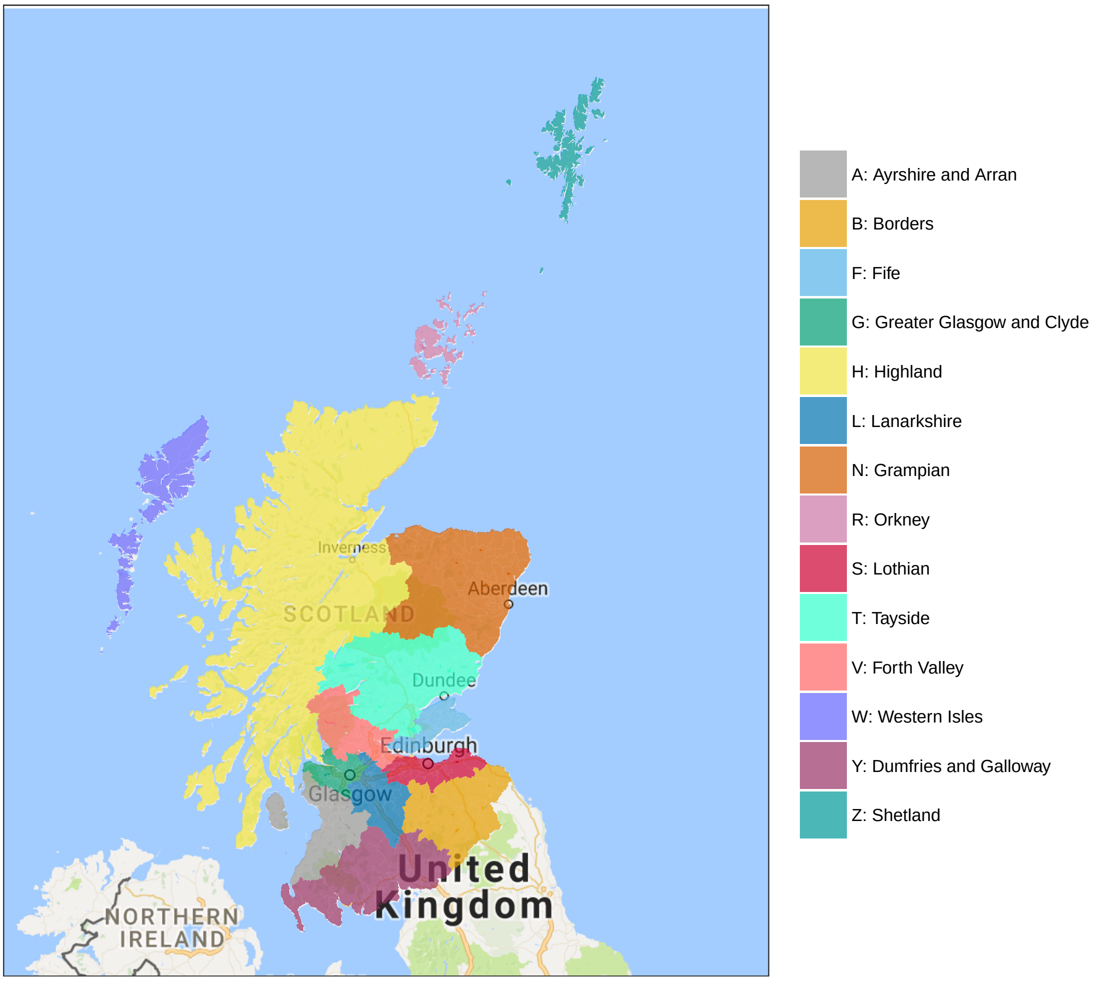

Fig. 1. Map of the NHS Health Boards in Scotland. Map data ©2017 Google.

example, for Lanarkshire, a decreasing trend can be seen for coronary heart disease, and in 2003 this disease has the highest estimated risk for Lanarkshire. However by the end of the time period, the median risk for this disease is lowest for this HB. A decreasing trend can also be seen for cerebrovascular disease. However, for respiratory disease an increasing trend can be seen, with the risk of this disease being the lowest of the three diseases in 2003 but the highest in 2012. In fact, a lot of the HB's show this switch in disease risk from coronary heart disease having the highest risk in 2003 to respiratory disease having the highest risk in 2012. For Western Isles, although all three diseases show a decreasing trend, the risk for coronary heart disease was much higher than for the other two diseases in 2003, and therefore the change in risk for this disease over time is much higher than for respiratory and cerebrovascular disease. Another feature of these plots is the increased variability in the estimates for the island HB's (Shetland, Western Isles and Shetland), which can be seen by the wider credible intervals and is due to them having the smallest numbers of IG's.

5. Intermediate geography risks

In order to investigate which IG's are at most risk of each disease, Table 2 shows the IG's with the top five highest risk estimates for each disease at the start of the time period (2003) and at the end (2012). Firstly, when comparing the IG's in 2003 to 2012 within disease, we notice that there are some IG's who make the top five in both these years. For example, in coronary heart disease three of the IG's with the highest risk in 2003 appear in the 2012 list, all of which belong to the Greater Glasgow and Clyde (G) HB. For respiratory disease, two IG's remain in the top five for both years, again both of which belong to Greater Glasgow and Clyde. Finally, all five IG's for cerebrovascular disease remain the same over the time period and all of these top five IG's belong to Greater Glasgow and Clyde. This tells us that the IG's which are at most risk of these diseases remain reasonably consistent over the time period. When we compare the IG's across the diseases we also notice some similarities. For example, IG Paisley Ferguslie which is in Renfrewshire not only appears in the top five highest risks for all diseases in both years, but actually comes out on top for coronary heart disease and respiratory disease and is second highest for cerebrovascular disease in both years. IG Easterhouse South (full name: North Barlanark and Easterhouse South) in Glasgow appears in the top five for coronary heart disease and cerebrovascular disease for both years. Finally, Drumchapel North in Glasgow appears for respiratory disease in 2012 as well as for cerebrovascular disease in both years. It should also be noticed that 24 of the 30 IG's that appear in Table 2 are IG's that belong to the Health Board Greater Glasgow and Clyde (G), which backs up the well known 'Glasgow effect'.

6. Comparison to other models

6.1. Temporally changing beta

The results from the model which allows the regression parameters to change over time can be found seperately for each disease in Tables 3, 4 and 5. In general these show little change in both the point estimates and the 95% credible intervals over time and compared

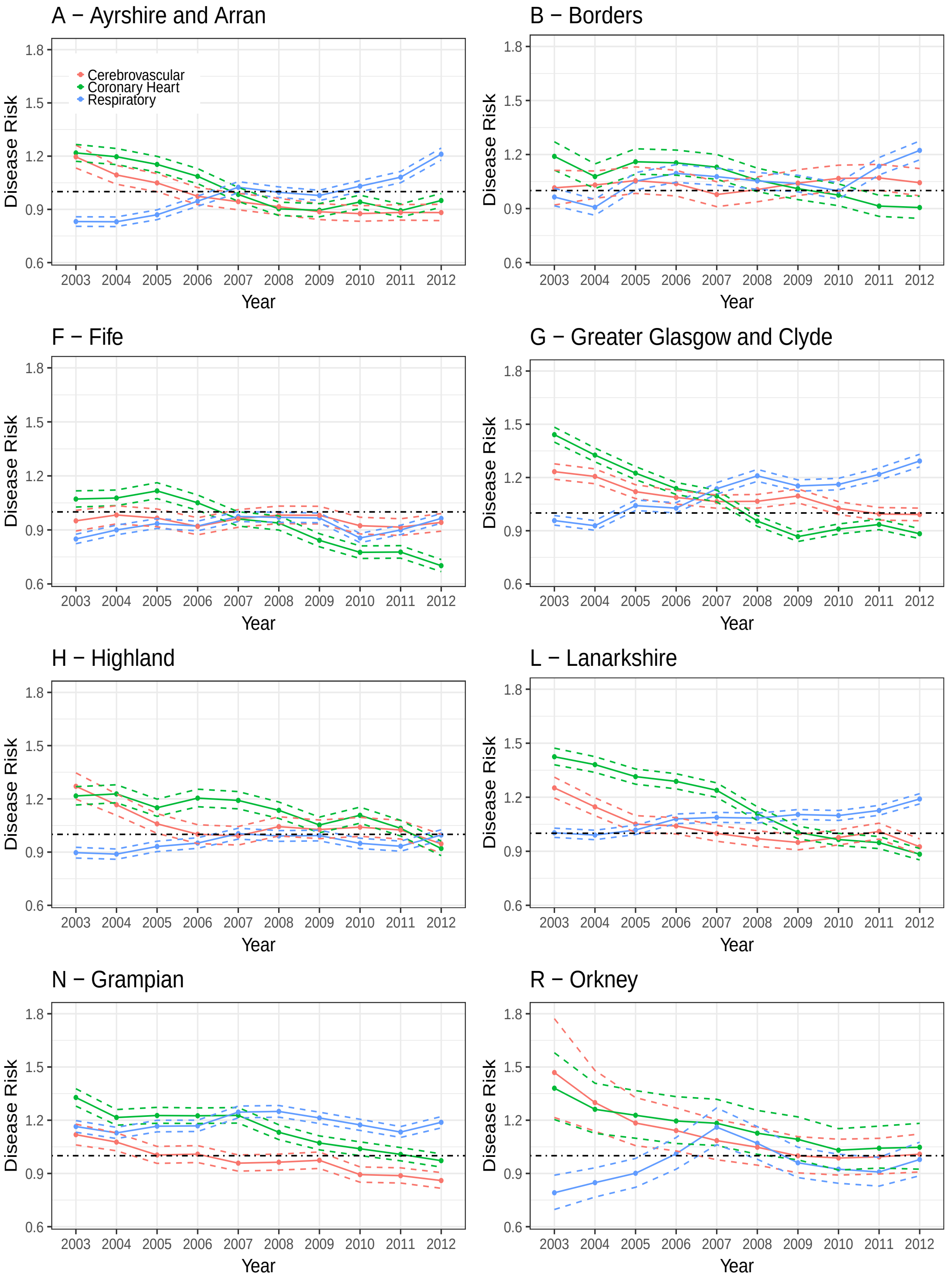

Fig. 2. Health Board risk effects across time $\left(R_{htd} = \frac{\sum_{i \in I_h} \pi_i \exp(\phi_{id} + HB_{htd})}{\sum_{i \in I_h} \pi_i}\right)$ for each disease. Posterior medians in red for cerebrovascular, green for coronary heart and blue for respiratory disease. Black dashed line indicates the null risk of 1 and 95% credible intervals shown by coloured dashed lines.

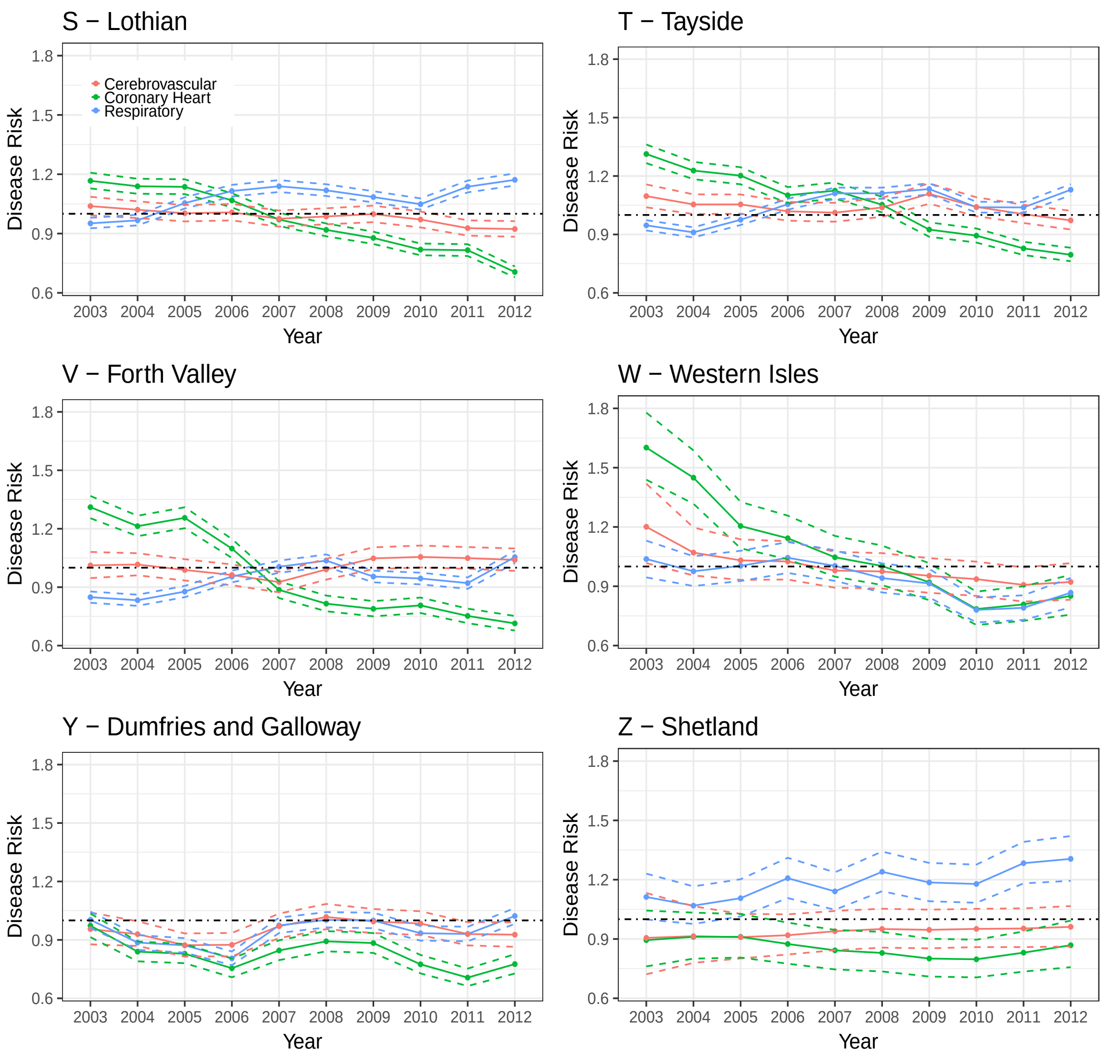

Fig. 3. Health Board risk effects across time $\left(R_{htd} = \frac{\sum_{i \in I_h} \pi_i \exp(\phi_{id} + HB_{htd})}{\sum_{i \in I_h} \pi_i}\right)$ for each disease. Posterior medians in red for cerebrovascular, green for coronary heart and blue for respiratory disease. Black dashed line indicates the null risk of 1 and 95% credible intervals shown by coloured dashed lines.

IG Code	HB	Median Risk	95% CI
**Coronary Heart Disease (2003)**			
Paisley Ferguslie	G	2.724	(2.454,3.024)
Lower Bow & Larkfield	G	2.498	(2.296,2.709)
Easterhouse South	G	2.435	(2.181,2.713)
Garthamlock	G	2.267	(2.025,2.521)
Braeside	G	2.256	(2.058,2.471)
**Coronary Heart Disease (2012)**			
Paisley Ferguslie	G	1.665	(1.497,1.849)
Inverness Merkinch	H	1.536	(1.355,1.732)
Lower Bow & Larkfield	G	1.526	(1.403,1.659)
Braehead	A	1.510	(1.371,1.665)
Easterhouse South	G	1.489	(1.331,1.661)
**Respiratory Disease (2003)**			
Paisley Ferguslie	G	1.955	(1.843,2.073)
Viewpark	L	1.848	(1.751,1.950)
Heathryfold	N	1.820	(1.713,1.930)
Drumry East	G	1.794	(1.677,1.918)
Greendykes	S	1.787	(1.667,1.913)
**Respiratory Disease (2012)**			
Paisley Ferguslie	G	2.627	(2.478,2.786)
Doon Valley South	A	2.507	(2.357,2.665)
Drumry East	G	2.412	(2.254,2.578)
Drumry West	G	2.394	(2.246,2.550)
Drumchapel North	G	2.327	(2.171,2.494)
**Cerebrovascular Disease (2003)**			
Easterhouse South	G	2.385	(2.092,2.717)
Paisley Ferguslie	G	2.226	(1.938,2.544)
Parkhead West	G	2.064	(1.864,2.291)
Drumchapel North	G	2.060	(1.755,2.410)
Parkhead North	G	2.011	(1.810,2.231)
**Cerebrovascular Disease (2012)**			
Easterhouse South	G	1.910	(1.677,2.178)
Paisley Ferguslie	G	1.781	(1.554,2.043)
Parkhead West	G	1.654	(1.492,1.837)
Drumchapel North	G	1.650	(1.405,1.934)
Parkhead North	G	1.612	(1.451,1.789)

Table 2: Posterior medians and 95% credible intervals for the top 5 IG's with the highest risks for the years 2003 and 2012 for each disease. The IG's which appear for more than one disease appear in colour.

with the corresponding estimates from the model with temporally static estimates. In almost all cases the 95% credible intervals overlap for all pairs of time periods

6.2. No covariates

In order to test the sensitivity of our results to the choice of covariates, our model was run again with no covariate effects. The results were virtually the same and some figures are shown here for comparison. Figure 4 shows boxplots of the disease risk for all three diseases from the model with no covariates. When compared to Figure 5 in the main text, it can be seen that there is very little change. Figure 5 in this supplementary material shows the fitted values from the model with covariates (x-axis) and the model without (y-axis) and the results are practically unchanged.

6.3. Multivariate spatio-temporal random effect

Figure 6 shows the disease risk for each of the diseases in IG's in Scotland across the time period using the results from the model proposed by Quick et al. (2017). When compared to the same plot using the results from our model (Figure 5 in main paper) it can be seen that these results are practically identical. This is backed up in Figure 7, which shows the fitted values from our model (M1) on the x-axis and the corresponding fitted values from the model proposed by Quick et al. (2017) (M2) on the y-axis. Again this shows that the results are very similar.

**Covariate - Cerebrovascular**	**Median RR**	**95% CI**
**% 16-64 year olds claiming JSA**		
2003	**1.063**	**(1.054, 1.072)**
2004	**1.061**	**(1.052, 1.069)**
2005	**1.065**	**(1.057, 1.074)**
2006	**1.059**	**(1.050, 1.067)**
2007	**1.058**	**(1.049, 1.067)**
2008	**1.061**	**(1.053, 1.070)**
2009	**1.059**	**(1.051, 1.068)**
2010	**1.056**	**(1.048, 1.065)**
2011	**1.056**	**(1.047, 1.065)**
2012	**1.060**	**(1.051, 1.068)**
**Log % Asian**		
2003	1.004	(0.980, 1.029)
2004	0.990	(0.967, 1.014)
2005	1.004	(0.979, 1.027)
2006	1.015	(0.992, 1.040)
2007	0.985	(0.962, 1.009)
2008	0.993	(0.970, 1.016)
2009	1.000	(0.981, 1.029)
2010	**0.995**	**(0.940, 0.987)**
2011	0.993	(0.968, 1.017)
2012	1.018	(0.994, 1.043)
**Log % Black**		
2003	1.005	(0.992, 1.017)
2004	**1.023**	**(1.010, 1.036)**
2005	**1.014**	**(1.002, 1.027)**
2006	1.002	(0.989, 1.014)
2007	**1.017**	**(1.002, 1.031)**
2008	1.001	(0.989, 1.013)
2009	1.008	(0.995, 1.021)
2010	**1.018**	**(1.006, 1.031)**
2011	1.009	(0.996, 1.022)
2012	0.992	(0.980, 1.004)
**Rural area**		
2003	1.036	(0.978, 1.098)
2004	1.009	(0.954, 1.068)
2005	0.997	(0.940, 1.053)
2006	**0.938**	**(0.888, 0.992)**
2007	0.958	(0.903, 1.011)
2008	0.960	(0.910, 1.013)
2009	0.993	(0.940, 1.046)
2010	0.950	(0.902, 1.006)
2011	1.004	(0.950, 1.060)
2012	**0.940**	**(0.887, 0.994)**

Table 3: Relative risk estimates for a 1% increase in each covariate (not urban/rural covariate) and 95% credible intervals for the covariates in a model with temporally varying regression parameters for cerebrovascular disease. Significant results are in bold.

**Covariate - Coronary Heart Disease**	**Median RR**	**95% CI**
**% 16-64 year olds claiming JSA**		
2003	**1.067**	**(1.059, 1.074)**
2004	**1.067**	**(1.060, 1.075)**
2005	**1.063**	**(1.055, 1.070)**
2006	**1.058**	**(1.050, 1.065)**
2007	**1.059**	**(1.052, 1.067)**
2008	**1.069**	**(1.061, 1.076)**
2009	**1.066**	**(1.059, 1.074)**
2010	**1.065**	**(1.057, 1.073)**
2011	**1.063**	**(1.055, 1.071)**
2012	**1.067**	**(1.061, 1.077)**
**Log % Asian**		
2003	**0.944**	**(0.927, 0.962)**
2004	**0.953**	**(0.936, 0.971)**
2005	**0.932**	**(0.916, 0.950)**
2006	**0.944**	**(0.927, 0.962)**
2007	**0.961**	**(0.944, 0.980)**
2008	**0.977**	**(0.960, 0.998)**
2009	1.002	(0.983, 1.010)
2010	0.989	(0.970, 1.009)
2011	**0.974**	**(0.956, 0.995)**
2012	0.985	(0.966, 1.007)
**Log % Black**		
2003	1.003	(0.994, 1.013)
2004	1.003	(0.994, 1.013)
2005	0.999	(0.989, 1.008)
2006	0.996	(0.986, 1.005)
2007	1.000	(0.991, 1.010)
2008	0.997	(0.988, 1.007)
2009	1.000	(0.990, 1.009)
2010	1.006	(0.996, 1.017)
2011	1.004	(0.994, 1.015)
2012	1.012	(1.002, 1.022)
**Rural area**		
2003	**0.920**	**(0.881, 0.965)**
2004	**0.942**	**(0.901, 0.984)**
2005	**0.911**	**(0.871, 0.955)**
2006	**0.942**	**(0.900, 0.984)**
2007	**0.954**	**(0.913, 0.997)**
2008	0.968	(0.925, 1.012)
2009	0.978	(0.935, 1.024)
2010	0.989	(0.945, 1.034)
2011	0.980	(0.934, 1.028)
2012	0.987	(0.942, 1.033)

Table 4: Relative risk estimates for a 1% increase in each covariate (not urban/rural covariate) and 95% credible intervals for the covariates in a model with temporally varying regression parameters for coronary heart disease. Significant results are in bold.

**Covariate - Respiratory**	**Median RR**	**95% CI**
**% 16-64 year olds claiming JSA**		
2003	**1.100**	**(1.093, 1.078)**
2004	**1.101**	**(1.094, 1.110)**
2005	**1.104**	**(1.097, 1.113)**
2006	**1.101**	**(1.095, 1.109)**
2007	**1.101**	**(1.094, 1.109)**
2008	**1.108**	**(1.100, 1.116)**
2009	**1.108**	**(1.101, 1.116)**
2010	**1.104**	**(1.098, 1.113)**
2011	**1.104**	**(1.097, 1.112)**
2012	**1.106**	**(1.100, 1.114)**
**Log % Asian**		
2003	**0.970**	**(0.953, 0.988)**
2004	0.986	(0.969, 1.005)
2005	0.987	(0.971, 1.006)
2006	0.983	(0.966, 1.001)
2007	**0.977**	**(0.960, 0.995)**
2008	**0.979**	**(0.961, 0.996)**
2009	1.000	(0.982, 1.017)
2010	0.995	(0.978, 1.013)
2011	**0.976**	**(0.960, 0.994)**
2012	**0.981**	**(0.963, 0.999)**
**Log % Black**		
2003	0.999	(0.990, 1.008)
2004	0.997	(0.988, 1.005)
2005	0.993	(0.985, 1.001)
2006	0.998	(0.990, 1.006)
2007	0.997	(0.989, 1.004)
2008	0.992	(0.984, 1.000)
2009	**0.987**	**(0.979, 0.995)**
2010	**0.991**	**(0.983, 0.999)**
2011	0.992	(0.984, 1.000)
2012	**0.988**	**(0.980, 0.996)**
**Rural area**		
2003	0.993	(0.957, 1.040)
2004	1.011	(0.970, 1.055)
2005	1.015	(0.977, 1.058)
2006	1.027	(0.989, 1.072)
2007	0.991	(0.954, 1.030)
2008	0.968	(0.971, 1.051)
2009	0.978	(0.987, 1.069)
2010	0.989	(0.936, 1.010)
2011	0.980	(0.927, 1.000)
2012	0.987	(0.964, 1.041)

Table 5: Relative risk estimates for a 1% increase in each covariate (not urban/rural covariate) and 95% credible intervals for the covariates in a model with temporally varying regression parameters for respiratory disease. Significant results are in bold

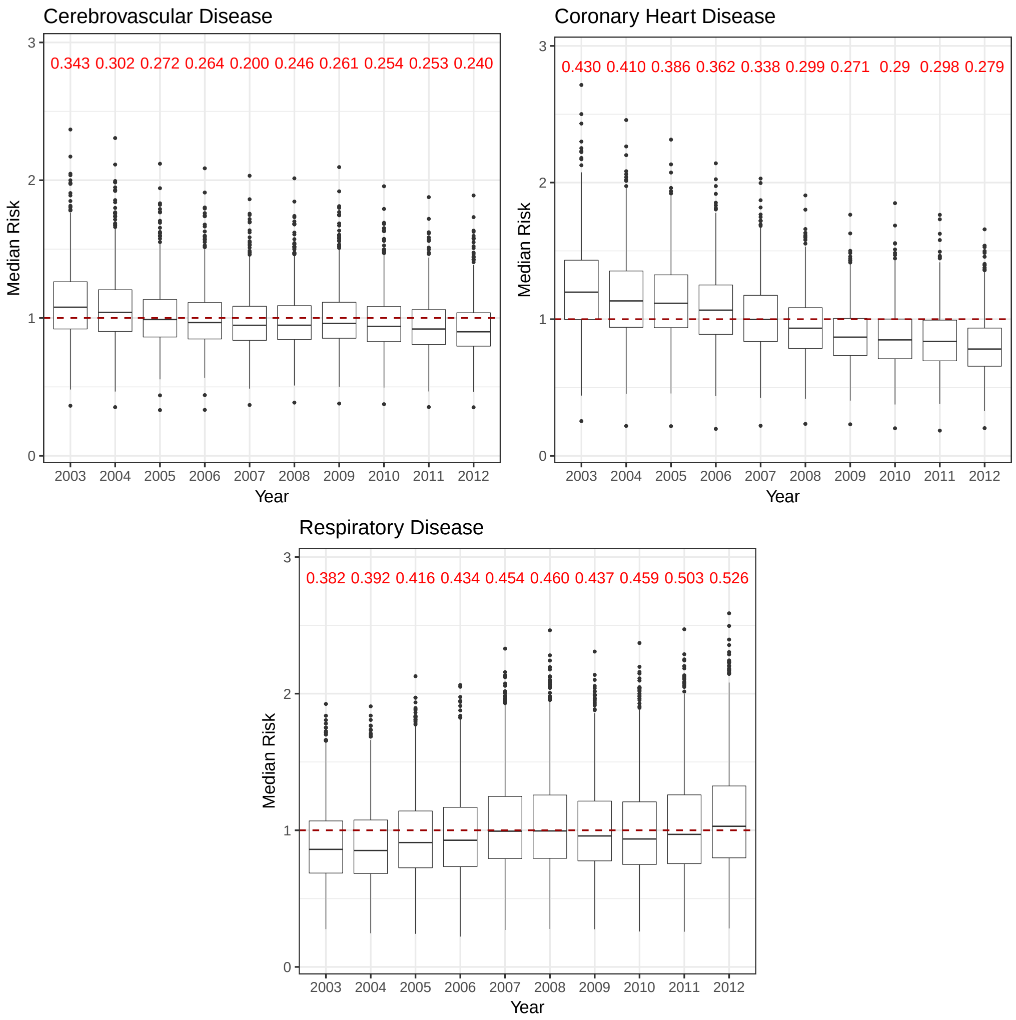

Fig. 4. Boxplots of disease risk for a model without covariates for cerebrovascular disease, coronary heart disease, and respiratory disease in IG's in Scotland from 2003 - 2012. The IQR across IG's are printed in red. Outliers are those observations that lie outside 1.5*IQR.

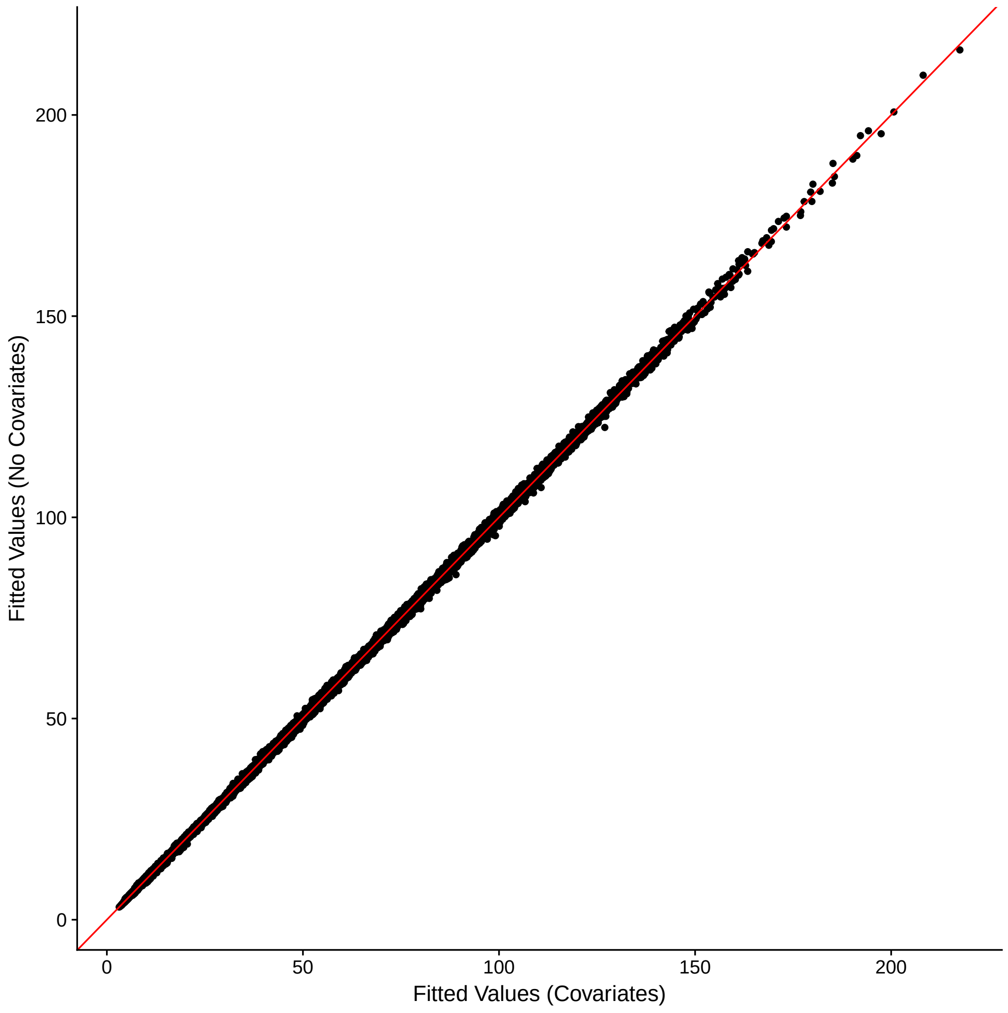

Fig. 5. Scatterplot of fitted values from model with covariates vs fitted values from model without covariates.

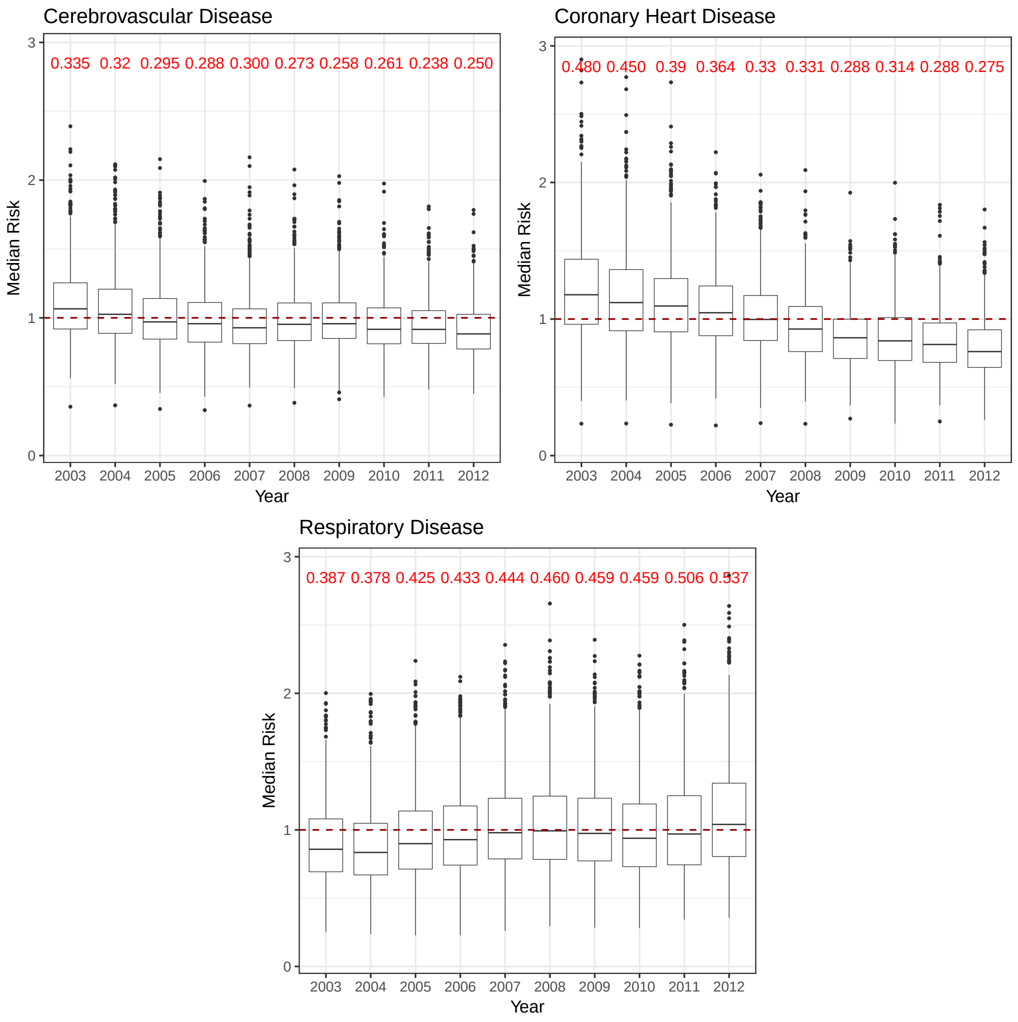

Fig. 6. Boxplots of disease risk from the Quick et al. (2017) model for cerebrovascular disease, coronary heart disease, and respiratory disease in IG's in Scotland from 2003 - 2012. The IQR across IG's are printed in red. Outliers are those observations that lie outside 1.5*IQR.

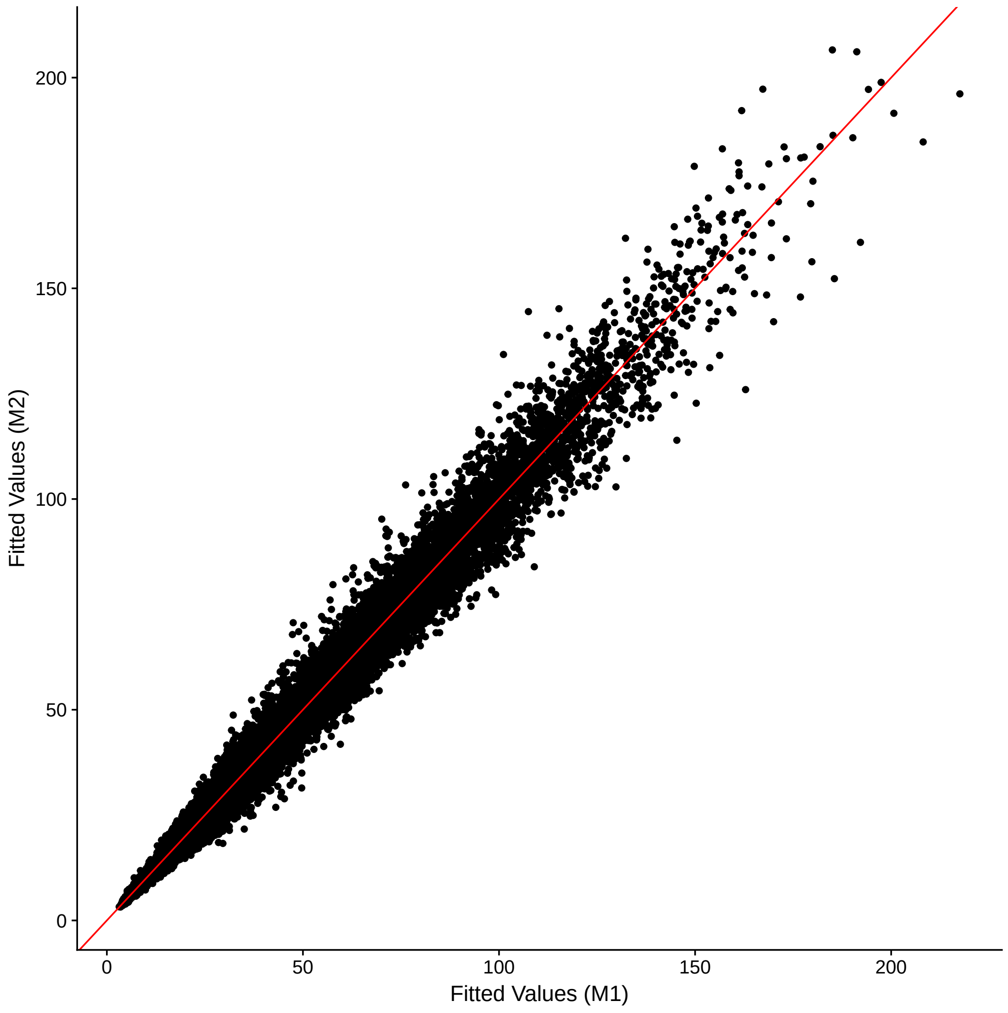

Fig. 7. Scatterplot of fitted values from our model (M1) vs fitted values from the Quick et al. (2017) model (M2).
