# Supplementary material for: Estimating the changing nature of Scotland's health inequalities by using a multivariate spatiotemporal model
Source: J R Stat Soc Ser A Stat Soc. 2019 Apr 9;182(3):1061–80. doi: 10.1111/rssa.12447 (PMC6563432; doi:10.1111/rssa.12447)
Supplement: Supplementary file 3 [file RSSA-182-1061-s003.pdf]

Coronary Heart Disease

Eilidh Jack, Duncan Lee, Nema Dean

1. SIR map of coronary heart disease

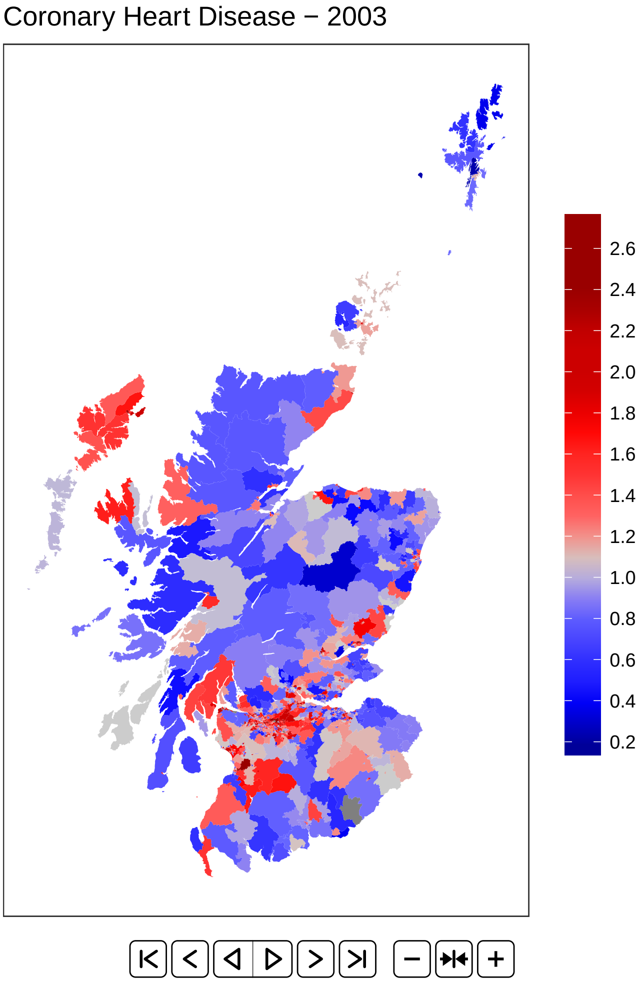

Fig. 1. Standardised Incidence Ratios (SIR) for coronary heart disease for each IG in Scotland from 2003 to 2012.
